# Geniposide Inhibits Oral Squamous Cell Carcinoma by Regulating PI3K-Akt Signaling-Mediated Apoptosis: A Multi-Method Validation Study

**DOI:** 10.3390/cimb47090786

**Published:** 2025-09-22

**Authors:** Xue Wang, Jianbo Wang, Hua Hua, Ping Wei, Xue Chen, Yusheng Peng, Li Liu, Dongmei Yu, Xiaozhou You, Siye Yang

**Affiliations:** Sichuan Institute for Translational Chinese Medicine, Sichuan Academy of Chinese Medicine Sciences, Translational Chinese Medicine Key Laboratory of Sichuan Province, Chengdu 610000, China

**Keywords:** geniposide, OSCC, network pharmacology, molecular docking, molecular dynamics simulation, apoptosis, PI3K-Akt signaling pathway

## Abstract

*Gardenia jasminoides* J.Ellis is an important medicinal and edible resource. The fruit of *Gardenia jasminoides* J.Ellis contains a natural iridoid called geniposide, which has the ability to dramatically suppress the growth of a number of cancer cell lines. This work examined the impact and potential mechanism of action of geniposide on oral squamous cell carcinoma using network pharmacology, molecular docking, molecular dynamics simulation, and cellular experiments. Based on network pharmacology, 145 potential targets of geniposide in the treatment of OSCC were found. The top five core targets were selected according to the degree values of the nodes, AKT1, EGFR, SRC, HSP90AA1, and PIK3R1, which involved signaling pathways and biological processes, such as the PI3K-Akt signaling pathway, pathways in cancer, phosphorylation, and the regulation of the apoptotic process. Molecular docking showed that geniposide exhibited good binding ability with the core targets AKT1 and EGFR. Molecular dynamics simulations further confirmed the stability of the binding between geniposide and the targets. The results of cell experiments showed that the activity of HSC-3 cells was dose-dependently inhibited by geniposide, and AO/EB staining showed that geniposide was able to induce programmed apoptosis. Meanwhile, it was found that the expressions of p-EGFR, p-AKT, and Bcl-2 were downregulated in HSC-3, and the expressions of PTEN, Bax, and Caspase-3 were upregulated. Geniposide may inhibit OSCC by affecting the PI3K-Akt signaling pathway and apoptotic process by regulating the expressions of p-EGFR, p-AKT, Bcl-2, Bax, Caspase-3, and PTEN.

## 1. Introduction

Oral cancer (OCC) is a type of head and neck cancer (HNC), and more than 90% of OCC originates from squamous tissues, called oral squamous cell carcinoma (OSCC) [1], which originates from the lining of the oral cavity, usually on the tongue, lips, and floor of the mouth [2]. OSCC is the most common malignant tumor of the oral and maxillofacial region, with morbidity and mortality rates ranking among the top 10 cancers in the spectrum of cancer, with a continuously rising trend [3]. Due to primary dental clinics’ and the general public’s lack of experience in identifying and treating the early symptoms of OSCC, more than 60% of patients are diagnosed with OSCC in the intermediate to advanced stages (Stage III/IV), which has a substantial impact on the survival rate and therapeutic efficacy [4]. Betel nut, alcohol, tobacco, daily nutrition, and lifestyle choices all have an impact on the complicated etiology of OSCC. Treatment and management face challenges [2,5]. Surgical resection, radiation, immunotherapy, and targeted therapy are currently the mainstays of treatment for OSCC; however, issues with medication resistance, patient adaptation, functional rehabilitation, and quality of survival still exist. Therefore, creating and using medications that are effective against OSCC is crucial.

**Figure 1:**
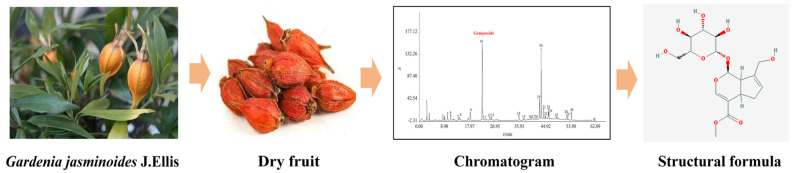
Source of geniposide.

Homology of medicine and food from natural sources, in which active ingredients are derived from plants or traditional Chinese herbs and have multiple targets, multiple mechanisms, and a variety of biological activities, including anti-inflammatory, anti-tumor, and antioxidant properties, and exhibit therapeutic efficacy and reduced toxicity and side effects in clinical studies, may offer special benefits when used to improve OSCC. The fruit of *Gardenia jasminoides* J.Ellis contains a natural iridoid called geniposide, which has anti-inflammatory, antioxidant, anticancer, antidiabetic, hepatoprotective, and immunomodulatory properties [6]. Regarding anticancer properties, geniposide has the ability to dramatically suppress the growth of a number of cancer cell lines, including oral squamous cell carcinoma cells [7,8], hepatocellular carcinoma cells [9], diffuse large B-cell lymphoma cells [10], medulloblastoma cells [11], gastric MKN45 cells [12], etc., mainly through the PI3K-Akt signaling pathway connected to apoptosis. Genipin, the primary active metabolite of geniposide, causes apoptosis, stimulates autophagy, and inhibits the activity of oral squamous cell carcinoma cells. The PI3K-Akt signaling pathway is intimately linked to its mechanism [13]. Therefore, we hypothesize that geniposide may also affect the PI3K-Akt signaling pathway as OSCC develops. Nevertheless, there is currently insufficient proof to substantiate this theory. In order to provide a new method and theoretical foundation for the treatment of OSCC, we examined the impact and potential mechanism of action of geniposide on this disease using network pharmacology, molecular docking, molecular dynamics simulation, and cellular experiments (Figure 1).

## 2. Materials and Methods

### 2.1. Materials

#### 2.1.1. Cell Strain

Oral squamous cell carcinoma cell line (HSC-3) (Shanghai Fuheng Biotechnology Co., Ltd., Shanghai, China, No. FH1315) was used.

#### 2.1.2. Drugs and Reagents

The following drugs and reagents were used: geniposide (Chengdu Aiboke Biotechnology Co., Ltd., Chengdu, China, (mass fraction ≥ 98%), No. AB1226), MEM basic (1X), fetal bovine serum (Wuhan Pricella Biotechnology Co., Ltd., Wuhan, China, No. PM150410, 164210-50), Penicillin–Streptomycin, Trypsin 0.25% (1X) solution (Thermo Fisher Scientific HyClone, Waltham, MA, USA, No. SV30010, SH30042.01), cytotoxicity detection and MTT cell proliferation kit, SDS-PAGE (Beyotime Biotechnology Co., Ltd., Shanghai, China, No. C0009S, P0012AC), phosphate-buffered saline (PBS) (Wuhan Servicebio Technology Co., Ltd., Wuhan, China, No. G4202), AO/EB double fluorescence staining kit (Beijing Bioss Biotechnology Co., Ltd., Beijing, China, No. S0012), RIPA, PMSF, BCA protein concentration kit (Ranjeck Technology Co., Ltd., Beijing, China, No. BL504A, BL507A, BL521A), phosphatase inhibitor (Wuhan Servicebio Technology Co., Ltd., Wuhan, China, No. G2007-1ML), PVDF (Bio-Rad Laboratories, Inc., Hercules, CA, USA, No. 1620177), EGFR, AKT, p-EGFR, p-AKT, PTEN, Caspase-3 (Proteintech Group, Inc., Wuhan, China, No. 66455-1-Ig, 30277-1-AP, 10176-2-AP, 66444-1-Ig, 60300-1-Ig, 25128-1-AP), Bcl-2, β-actin, Bax (Affinity BioReagents, Inc., Golden, CO, USA, No. AF6139, AF7018, AF0120), and HRP-labeled Goat Anti-Rabbit IgG (Beijing Zhongshan Golden Bridge Biotechnology Co. Ltd., Beijing, China, No. ZB-2301).

#### 2.1.3. Instruments

The following instruments were used: incubator (PHC Holdings Corporation, Tokyo, Japan, MCO-18AC), Inverted Microscope (Guangzhou Mshot Photoelectric Technology Co., Ltd., Guangzhou, China, M152-N), Analytical Balance (Shimadzu, Kyoto, Japan, AUY120), Benchtop Microcentrifuge (Scilogex, Rocky Hill, CT, USA, D3024), Multimode Reader (Tecan, Männedorf, Switzerland, Spark CN), and E-Gel Imager (Clinx, Shanghai, China, ChemiScope6100).

### 2.2. Methods

#### 2.2.1. Network Pharmacology, Molecular Docking, and Molecular Dynamics Simulation

##### Geniposide’s Molecular Structure and Target Gene Screening

The PubChem database was searched with the keyword “geniposide” to acquire the chemical structure of geniposide. The potential targets of geniposide were identified using the Pharmmapper and SwissTargetPrediction databases (https://www.lilab-ecust.cn/pharmmapper/ and http://swisstargetprediction.ch/, URL (accessed on 13 May 2024)), and the targets were screened according to Norm Fit > 0 and probability > 0. The targets were converted to unified gene names by the Uniprot database (https://www.uniprot.org/, URL (accessed on 13 May 2024)), merged, and de-emphasized to obtain the potential targets of geniposide.

##### OSCC Target Gene Acquisition

The keyword “Oral Squamous Cell Carcinoma” was used to gather OSCC-related targets from the OMIM and GeneCards databases (https://www.omim.org/ and https://www.genecards.org/, URL (accessed on 13 May 2024)). The GeneCards score was set to ≥20, and the database results were pooled and de-weighted to obtain potential targets.

##### Drug–Disease Cross-Target Screening and Drug–Target–Disease Network Construction

The common target genes of geniposide and OSCC were identified by intersecting the screened geniposide targets with genes linked to OSCC disease using the Venny2.1.0 platform. The mutual correspondence of “geniposide–target” and “OSCC–target” was imported into Cytoscape 3.8.2 software to create a “geniposide–targets–oral squamous cell carcinoma” network diagram.

##### Protein–Protein Interaction (PPI) Network Construction

The common target genes were imported into the String11.5 (www.string-db.org/, URL (accessed on 15 May 2024)) database, the species was set as “Homo sapiens”, the confidence level was >0.7, and the remaining value was the system default. The result file was imported into Cytoscape 3.8.2 software for visualization, the PPI network graph was created, free nodes were removed, and the top 5 targets were filtered based on node degree, node median, and node tightness as core targets.

##### GO and KEGG Analyses

The 145 intersecting targets were imported into the David database (https://davidbioinformatics.nih.gov/, URL (accessed on 15 May 2024)) for KEGG pathway analysis and GO function annotation.

##### Molecular Docking

Geniposide and the five targets in the PPI network with the highest degree values were molecularly docked. The RCSB PDB database (http://www.rcsb.org/, URL (accessed on 16 May 2024)) was used to screen protein targets and high-resolution crystal structures as receptors. PyMOL 2.5.0 software was used to dehydrate and dephosphorylate the protein and save it as a PDB file. The receptors underwent additional hydrogenation and dehydrogenation using AutoDock 4.2.6 software, molecular docking using AutoDock Vina 1.5.6 software [14], calculation of the ligand–receptor binding capacity, and visualization using Pymol and Discovery Studio 2019 software [15].

##### Molecular Dynamics Simulation

GROMACS2022.5 software [16] was used to conduct molecular dynamics simulations of the docking coordination complex between geniposide and targets. The topology generation used the AMBER99SB-ILDN force field for the protein to better describe side-chain conformations and nucleic acid stability [17,18,19]. For the ligand, a topology file was generated with ACPYPE using the GAFF force field, and its atomic charges were calculated with the AM1-BCC method. These steps ensured compatibility with the AMBER force field and maintained parameter consistency [20,21,22]. The energy was minimized using the steepest descent algorithm, and equilibrium was achieved by running the NVT and the NPT for 100 ps. The system was subsequently simulated for 100 ns. The Amber Tools software 23 package’s MMPBSA.py script was used to analyze the simulation system’s binding free energy. The interaction between each protein residue and ligand was predicted by MD sampling using MMPBSA (Poisson–Boltzmann surface area of molecular mechanics) based on the solvent accessibility method and the force field of molecular mechanics. Equation (1) was used to calculate the total binding free energy. Equation (2) was used to determine the free energy of the protein-ligand complex [23,24]. The simulation data were visualized by Xmgrace software 5.1.25.

(1) $$∆G_{bind}=G_{complex}-(G_{protein}+G_{ligand})$$

(2) $$∆G_{bind}=∆E_{MM}+∆G_{sol}-T∆S\;\;\;∆E_{MM}=∆E_{vdw}+∆E_{ele}$$

∆G_bind_ represents the free energy of binding, G_complex_ represents the free energy of the complex, and G_protein_ and G_ligand_ represent the free energy of the separated protein and ligand. ΔE_MM_ represents the gas phase (under vacuum) interaction energy between the target protein and ligand, which includes electrostatic interaction ΔE_ele_ and van der Waals ΔE_vdw_. ∆G_sol_ represents the solvation free energy difference, T∆S represents the entropy change in the ligand binding conformation at T temperature. However, T∆S has not been calculated in this study because it takes quite a long time to calculate configuration entropy, and it does not notably impact the contribution differences of the amino acids.

#### 2.2.2. Cell Experiments

##### Cell Culture and Drug Preparation

Oral squamous cell carcinoma cells (HSC-3) were expanded under complete medium at 37 °C, 5% CO_2_, and 98% humidity and with 10% fetal bovine serum, and the cells were passaged when the density reached 80%. Geniposide was dissolved in dimethyl sulfoxide (DMSO) to obtain a 10 mmol/L stock solution, which was diluted to concentrations of 0, 0.2, 0.4, 0.8, 1.0, 2.0, 2.4, and 2.8 mmol/L according to the experimental settings.

##### MTT Assay

HSC-3 cells in the logarithmic growth phase were seeded at 3 × 10^3^ cells per well in 96-well plates. They were incubated for 24 h at 37 °C with 5% CO_2_. Geniposide was added to the 96-well plates at the following concentrations: 0 (control), 0.2, 0.4, 0.8, 1.0, 2.0, 2.4, and 2.8 mmol/L. After 48 h, 20 μL of MTT solution was added, and the wells were incubated for 4 h. The supernatant was discarded, and 150 μL of DMSO was added to each well. The absorbance (A) value of each well at 490 nm was measured using an enzyme marker, and the IC50 and cell survival rate were computed. Three independent experiments were conducted, each with six replicate wells per concentration.

##### Acridine Orange/Ethidium Bromide (AO/EB) Staining

HSC-3 cells in the logarithmic growth phase were seeded at 1 × 10^6^ cells per well in 6-well plates with sterile coverslips and incubated at 37 °C in a 5% CO_2_ incubator for 24 h. The cells were treated with an IC50 concentration of geniposide for 24 h. Slides containing 30 μL of cell cultures were stained with AO/EB. The slides were covered with coverslips and photographed under a fluorescence microscope at five randomly selected locations. The number of living cells (VN), early apoptotic cells (VA), non-viable apoptotic cells (NVA), and necrotic cells (NVN) in each group was counted, and the apoptosis index (AI) was calculated.

(3) $$\mathrm{Apoptosis}\;\mathrm{index}\;(\%)=\frac{\mathrm{VA}+\mathrm{NVA}}{\mathrm{VN}+\mathrm{VA}+\mathrm{NVA}+\mathrm{NVN}}\;\times \;100\%\;$$

##### Western Blot

HSC-3 cells in the logarithmic growth phase were seeded at 1 × 10^6^ cells per well in 6-well plates with sterile coverslips and treated with geniposide at an IC50 concentration for 24 h. A blank control group was set up. Following collection and three PBS rinses, the total cellular proteins were extracted using RIPA with PMSF and phosphorylated protease inhibitor. The protein concentration was then determined using the BCA protein concentration assay kit. The proteins were separated by SDS-PAGE, transferred to a PVDF membrane, sealed with 5% skim milk powder for 30 min, and incubated with the appropriate primary antibodies against EGFR, p-EGFR, PTEN, AKT, p-AKT, Bcl-2, Bax, Caspase-3, and β-actin at 4 °C in a shaking bed overnight. The membrane was then quickly washed three times with TBST the following day (5 min), incubated with the matching secondary antibody at room temperature for 30 min, and finally washed three times with TBST for 5 min. The protein bands were detected in the developer solution, and the gray value of the bands was analyzed by Image J software 1.8.0.

##### Statistical Analysis

SPSS 22.0 and GraphPad Prism 8.0 software packages were used for statistical analysis. The data results are expressed as the mean ± SD. One-way analysis of variance (ANOVA) was used to analyze the data between multiple groups, the independent samples t-test was used to evaluate the data between two groups, and the statistics were statistically significant when *p* < 0.05.

## 3. Results

### 3.1. Prediction of Potential Targets of Geniposide and OSCC

**Figure 2:**
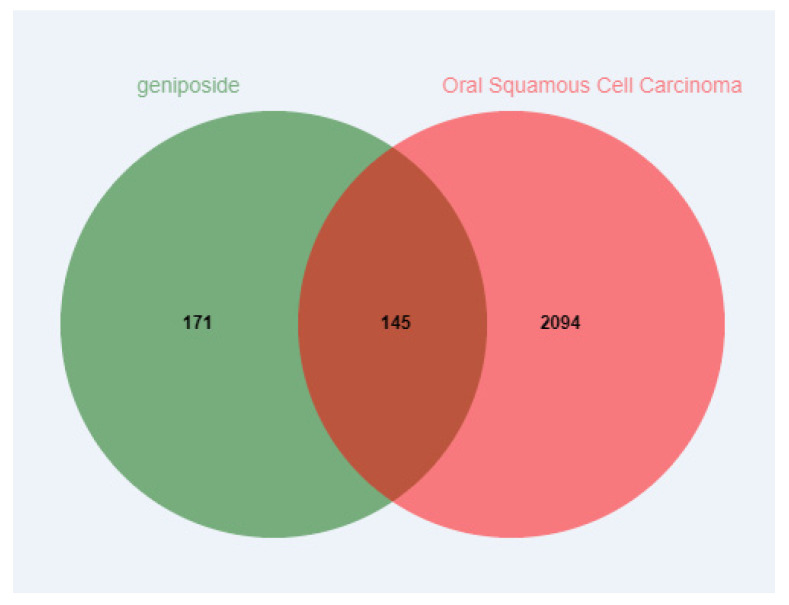
Venn diagram of intersection targets of geniposide and oral squamous cell carcinoma.

The potential target genes of geniposide were predicted by the SwissTargetPrediction (probability > 0) and Pharmmapper databases (probability > 0); duplicate targets were removed, and 316 targets were obtained. The GeneCards and OMIM databases were used to predict potential targets of OSCC; duplicate targets were removed, and 2239 targets were obtained. Mapping geniposide and OSCC targets to obtain intersection targets, a total of 145 targets were obtained (Figure 2).

### 3.2. “Drug–Target–Disease” Network Analysis

**Figure 3:**
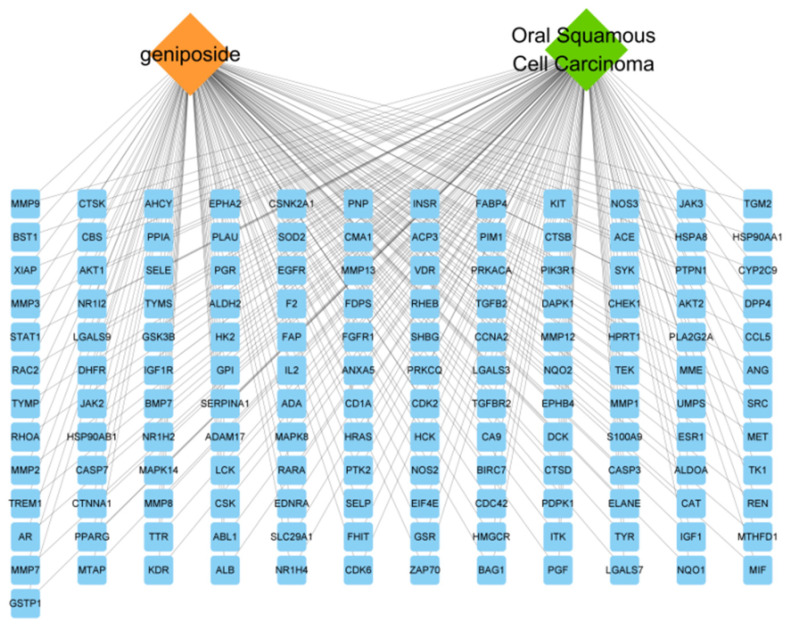
“Geniposide–targets–oral squamous cell carcinoma” network diagram.

The “geniposide–target–OSCC” network was constructed by Cytoscape3.8.2 software, using Network Analyzer to analyze the network; the results show that there are 145 nodes and 290 edges (Figure 3). The yellow rhombus represents geniposide, the green rhombus represents OSCC disease, and the blue rectangle represents the targets, indicating that the effect of geniposide on OSCC is a complex, synergistic regulation of multiple targets.

### 3.3. PPI Network Construction and Core Target Screening

**Figure 4:**
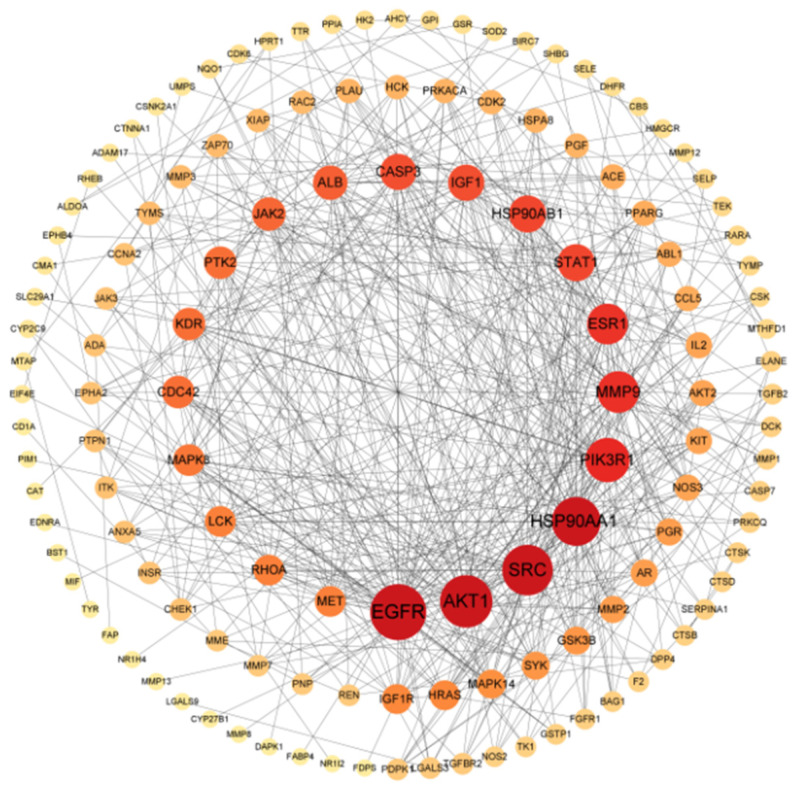
Construction of PPI network.

The potential targets of geniposide for OSCC were inputted into the String database to construct a PPI network. The result showed that there were 132 nodes and 541 edges (Figure 4), with an average degree of 8.197, node tightness of 0.343, and node mediativity of 0.016. The targets with degree values ranked in the top 10 were potential high-confidence target genes of geniposide for OSCC (Table 1). The top five degree values of EGFR, AKT1, SRC, HSP90AA1, and PIK3R1 were 44, 40, 38, 36, and 31, respectively.

### 3.4. GO Analysis and KEGG Signaling Pathway Analysis

**Figure 5:**
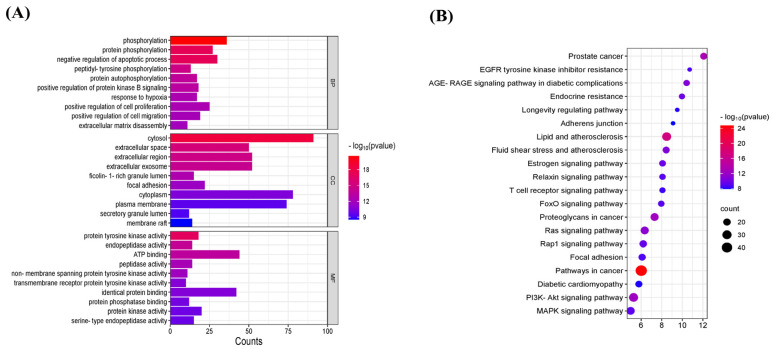
GO function and KEGG signaling pathway analysis. (**A**) GO function analysis. (**B**) KEGG signaling pathway analysis.

The 145 potential targets of geniposide for OSCC were functionally enriched and visualized. The molecular functions (MFs) in GO analysis included ATP binding, identical protein binding, etc.; the biological processes (BPs) included the regulation of the apoptotic process, phosphorylation, etc.; and the cellular component (CC) included the plasma membrane, cytosol, and extracellular exosome, etc. KEGG pathway analysis showed 145 potential targets involved in pathways in cancer, the PI3K-Akt signaling pathway, etc. (Figure 5A,B).

### 3.5. Molecular Docking

**Figure 6:**
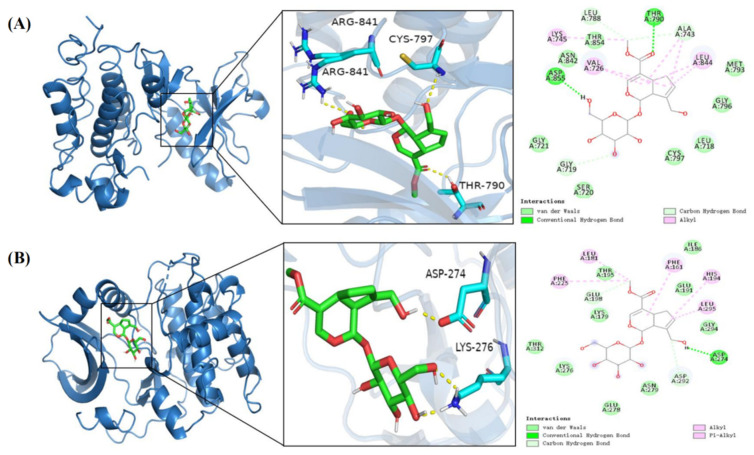
Molecular docking model of geniposide and EGFR and AKT-1 target proteins. (**A**) Geniposide–EGFR. (**B**) Geniposide–AKT-1.

In molecular docking of geniposide with the top five key targets, the smaller the binding energy, the higher the binding activity and the better the binding stability. A binding energy of less than −5.0 kcal/mol indicates potential binding, and a binding energy of less than −7.0 kcal/mol indicates strong binding stability [25]. Geniposide showed strong binding stability with EGFR with a binding energy of −8.1 kcal/mol and potential binding affinity to AKT1 with a binding energy of −6.8 kcal/mol. Further, the MET793, GLY796, LEU718, CYS797, SER720, GLY721, ASN842, and THR854 residues on the EGFR receptor were able to form hydrogen bonding forces with geniposide, the LEU844, LYS745, VAL726, and ALA743 residues were able to form hydrophobic forces with geniposide, the THR790 and ASP855 residues were able to form van der Waals forces with geniposide, and the LEU788, ALA743, and GLU719 residues were able to form Carbon hydrogen bond forces with geniposide. The THR195, GLU198, LYS179, THR312, LYS276, GLU278, ASN279, GLU294, GLU291, and ILE186 residues on the AKT1 receptor were able to form hydrogen bonding forces with geniposide, the PHE161, PHE225, LEU181, HIS194, and LEU295 residues were able to form hydrophobic forces with geniposide, the ASP274 residue was able to form van der Waals forces with geniposide, and the SP292 residue was able to form Carbon hydrogen bonding forces with geniposide (Figure 6A,B). These results indicate that Gardenia glycosides can effectively bind to the core target through hydrogen bonding, hydrophobic interactions, van der Waals forces, and Carbon hydrogen bond forces.

### 3.6. Molecular Dynamics Simulation

**Figure 7:**
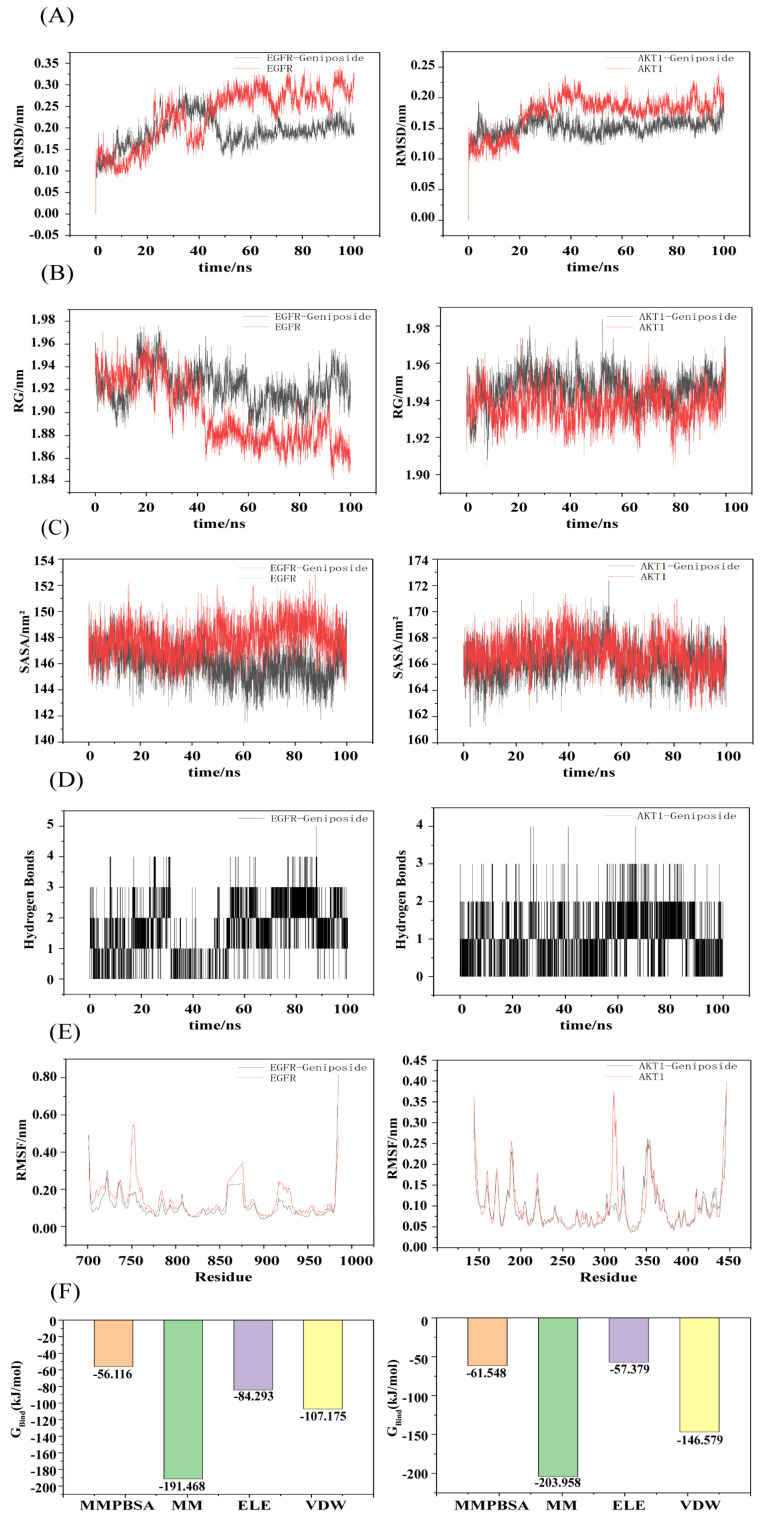
Molecular dynamics simulation of geniposide with core targets. (**A**) RMSD values of the two complexes. (**B**) Radius of gyration (Rg) values of the two complexes. (**C**) Solvent-accessible surface area (SASA) values of the two complexes. (**D**) Number of hydrogen bonds in the two complexes. (**E**) RMSF values of the two complexes. (**F**) Binding energies of the two complexes.

The root mean square deviation (RMSD) values of the complexes and the free-state ligand were used to determine whether or not the simulated system reached a steady state. RMSD values within 1 nm indicated that the complex structure was relatively stable. As shown in Figure 7A, the free-state EGFR ligand exhibited minor fluctuations within ±0.15 nm throughout the simulation. Following formation of the complex between geniposide and EGFR, a relatively stable state was attained after 50 ns, with the RMSD stabilizing within the 0.13–0.25 nm range and averaging 0.19 nm. The free AKT-1 ligand attained relative stability after 40 ns. Following formation of the complex between geniposide and AKT-1, a relatively stable state was attained after 30 ns, with the RMSD stabilizing within the 0.12–0.22 nm range, averaging 0.15 nm. These data indicate that both complexes exhibit stable binding. The radius of gyration (Rg) was evaluated for the tightness of the complex binding, and as shown in Figure 7B, it reveals that compared to the free protein, the Rg values of the geniposide complexes with both EGFR and AKT-1 exhibit relatively minor fluctuations and remain relatively stable throughout the simulation. This indicates that both complexes are tightly bound. The solvent-accessible surface area (SASA) is a crucial metric reflecting the tightness of complex binding. A smaller SASA value indicates less surface area exposed to the solvent and, thus, tighter binding between the target protein and small molecules. As shown in Figure 7C, the SASA values of the geniposide–EGFR complex gradually decreased over time compared to the free EGFR. These values exhibited relatively low measurements: 146.06 ± 1.36 nm and 147.85 ± 1.33 nm, respectively. The SASA values of the geniposide–AKT-1 complex were comparable to the free AKT-1 (166.22 ± 1.31 and 166.82 ± 1.34 nm, respectively) and showed similar fluctuation patterns. This further suggests that both complexes may possess high stability in the solvent environment. The number of hydrogen bonds reflects the stability of complex formation. As shown in Figure 7D, the hydrogen bond fluctuations for the geniposide complexes with EGFR and AKT-1 ranged between 0 and 5, and they had a high hydrogen bond density. These patterns suggest that the complexes exhibit interaction forces and possess a relatively stable structure. The root mean square fluctuation (RMSF) is used to determine the degree of fluctuation in residues during the simulation process. A lower RMSF value indicates that the structure of the region is more stable, while a higher RMSF value indicates that the residues have greater volatility. As shown in Figure 7E, comparing RMSF values between the geniposide–target protein complex and the free protein–ligand state reveals similar RMSF values for most residues. Following geniposide binding to EGFR, RMSF fluctuations at residues 750, 860, and 925 markedly decreased. Following formation of the geniposide–AKT-1 complex, RMSF fluctuations at residue 310 markedly decreased, indicating that the geniposide–target protein complex maintained a relatively stable conformation throughout the simulation [24,26,27]. The MM-PBSA method was used to calculate the binding energies of the two complexes, and a lower binding energy indicates stronger binding affinity [28]. The binding energies of the two complexes were −56.12 and −61.55 kJ/mol, respectively, indicating that geniposide binds strongly to EGFR and AKT-1 [24].

### 3.7. Geniposide’s Impact on OSCC Cell Activity

**Figure 8:**
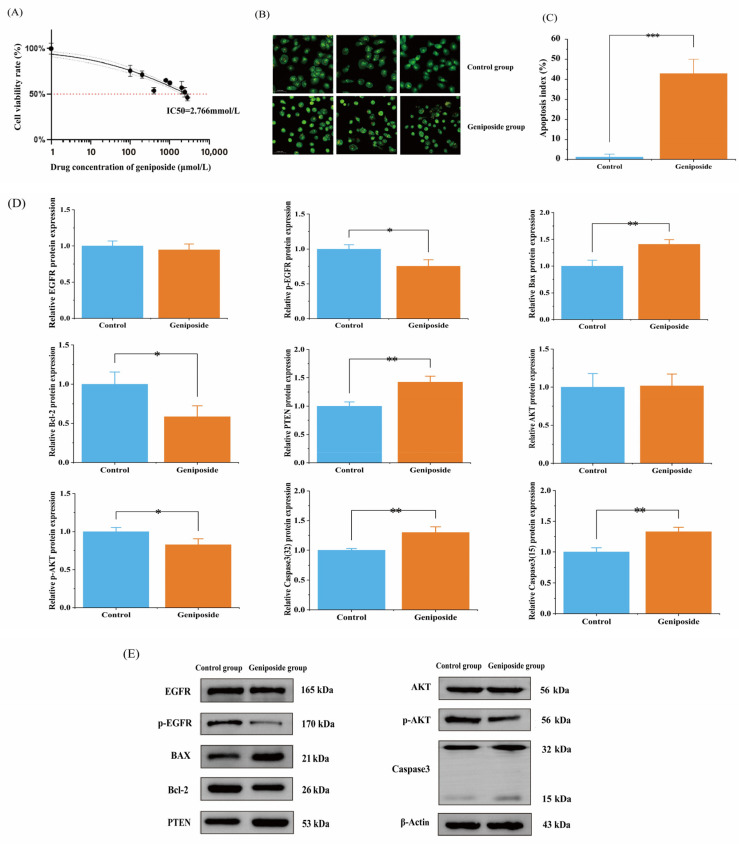
Results of cell experiments: (**A**) Effect of geniposide on the activity of HSC-3. (**B**) AO/EB staining. (**C**) Apoptosis index, *** *p* < 0.001 vs. control group. (**D**) HSC-3 cell protein expression rate, * *p* < 0.05 and ** *p* < 0.01 vs. control group. (**E**) Western blot results of HSC-3 cells.

After applying geniposide to HSC-3 cells at concentrations of 0.1, 0.2, 0.4, 0.8, 1.0, 2.0, 2.4, and 2.8 mmol/L for 48 h, the effects of the drug on OSCC (HSC-3) cell activity were measured using the MTT method. The results showed that geniposide could significantly inhibit HSC-3 cell activity (*p* < 0.01), there was a concentration correlation (Figure 8A), and the IC50 was 2.766 mmol/L.

### 3.8. Geniposide’s Impact on Apoptosis

The results of the AO/EB staining of HSC-3 cells showed that the IC50 concentration of geniposide triggered apoptosis in HSC-3 cells, while the control cells did not show signs of apoptosis. The apoptosis index values were 43.0 ± 0.072% and 1.0 ± 0.014%, respectively (*p* < 0.01) (Figure 8B,C).

### 3.9. Western Blot

Combining the results of the core targets, molecular docking, and signaling pathways, it was predicted that geniposide might interfere with OSCC through the PI3K-Akt signaling pathway and apoptosis-related pathway. Western blotting was used to determine how geniposide affected the expression of proteins. The results showed that after 24 h of geniposide action, the expression of PTEN, Bax, and Caspase-3 proteins was significantly increased (*p* < 0.01), the expression of p-EGFR, p-AKT, and Bcl-2 proteins was significantly decreased (*p* < 0.05), and the expression of EGFR and AKT proteins was not significantly changed (*p* > 0.05) (Figure 8D,E).

## 4. Discussion

OSCC is a serious threat to human health. It has a complex etiology, and its occurrence and development involve multiple targets and signaling pathways. The search for drugs and main therapeutic targets is of great significance for OSCC. Nowadays, traditional Chinese medicine monomers have obvious advantages in inhibiting tumor cell activity [29], and their multi-target properties can effectively alleviate tumor diseases. They also have low harmful side effects and are widely used in clinical research. Geniposide is a kind of iridoid, derived from the fruit of Gardenia jasminoides Ellis, a medicinal and edible plant, showing powerful effects on various tumor models in vitro and in vivo. A previous study found that geniposide had a significant destructive effect on the proliferation and invasion of cancer cells in mice with hepatocellular carcinoma [30]. It was also able to inhibit the proliferation and migration of the tongue squamous cell carcinoma (TSCC) line SCC-9 [7]. *Lactobacillus rhamnosus* GG strains and *Lactobacillus casei* strains were able to enhance the anticancer effects of geniposide in HSC-3 cells and increase the rate of apoptosis [8,31], but the target and the pathways they may affect are unclear.

In this study, based on network pharmacology, we screened the potential targets of geniposide against OSCC, obtained 145 intersecting targets, and constructed a “drug–target–disease” network, obtaining 145 nodes and 290 edges, which indicated that the effect of geniposide on OSCC is a complex and synergistic regulation of multiple targets. The PPI network analysis indicated that EGFR, AKT1, SRC, HSP90AA1, and PIK3R1 might be the key targets of geniposide for OSCC. In order to further verify the reliability of the results, the key targets were molecularly docked with geniposide, and the results showed that geniposide had strong binding stability with EGFR and AKT1, and residues on the receptors of EGFR and AKT1 were able to form hydrogen bonding forces, hydrophobic forces, van der Waals forces, and Carbon hydrogen bonds. The molecular dynamics simulation further confirmed the stability of the binding between geniposide and EGFR and AKT1 targets, indicating that EGFR and AKT1 may be crucial in the geniposide treatment of OSCC. Through GO function and KEGG signaling pathway analysis, the PI3K-Akt signaling pathway and apoptosis-related pathway were identified as the possible mechanism pathways of geniposide in the treatment of OSCC.

**Figure 9:**
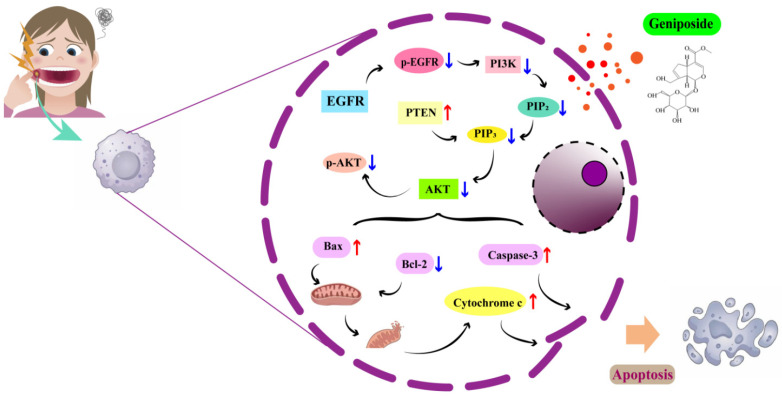
Mechanism diagram.

The PI3K-Akt signaling pathway, as a core regulatory hub of cellular function, plays a central role in physiological processes such as cell survival, proliferation, metabolism, and migration. Its abnormal activation has been directly linked to a number of serious illnesses, including cancer, diabetes, and neurodegenerative diseases [32]. Its activity is positively initiated by EGFR/p-EGFR and negatively regulated by PTEN, and it finely regulates Bcl-2/Bax balance and Caspase-3 activation through p-AKT, which determines cell survival. Under pathological circumstances, the system is abnormally activated because of EGFR mutation or overexpression, PTEN deletion, and PIK3CA mutation. This promotes proliferation and inhibits apoptosis, which drives cancer. EGFR, as an important member of the RTK family, plays the role of an initiation switch in the activation of the PI3K-Akt pathway. When EGFR binds to its ligand, it dimerizes and autophosphorylates to form activated p-EGFR. The phosphorylated tyrosine residues of p-EGFR can activate the lipid kinase activity of PI3K and the PI3K-Akt pathway. The aberrant overexpression or mutation of EGFR in a variety of solid tumors leads to the sustained activation of the PI3K-Akt pathway, which is a key driver in tumorigenesis and development [33,34]. Previous studies have found that the use of EGFR inhibitors can inhibit the proliferation, migration, and invasion of OSCC cells [35,36]. PTEN is a core negative regulator of the PI3K-Akt pathway, which plays a key role in the maintenance of signaling homeostasis. The deletion or functional inactivation of PTEN will sustain the activation of AKT and its downstream survival signals, which is an important mechanism for tumorigenesis [37,38]. A previous study found that PTEN expression gradually decreased in the successive steps of OSCC from normal to dysplastic to locally invasive to metastatic [39]. The inhibition of PTEN expression promotes the activation of the PI3K-AKT signaling pathway and induces malignant OSCC [40]. As an essential component of the PI3K-Akt signaling pathway, AKT plays a central role in signal transduction and downstream regulation. Through the phosphorylation of numerous downstream substrates, activated p-AKT regulates various biological processes including cell survival, proliferation, metabolism, and migration. In the regulation of cell survival, p-AKT effectively inhibits the apoptotic program through the triple mechanism of direct phosphorylation of the pro-apoptotic protein BAD, inhibition of Caspase-9 activation, and activation of NF-κB transcription factors (which promotes the expression of anti-apoptotic genes, such as Bcl-2) [41]. The PI3K-Akt pathway determines cell survival and death through the precise regulation of Bcl-2 family balance and Caspase activation. The pro-apoptotic (Bax) and anti-apoptotic (Bcl-2) members of the Bcl-2 family regulate mitochondrial outer membrane permeability through competitive dimerization. p-AKT activates the IKK-NF-κB pathway and promotes the transcription of Bcl-2. The AKT1 and Bcl-2 proteins are overexpressed in OSCC tissues and can promote cancer cell survival and proliferation [42,43] (Figure 9).

The results of this study showed that geniposide could reduce the proliferation of HSC-3 cells and cause apoptosis. Compared with the control group, after 24h of geniposide action, the expression of the PTEN, Bax, and Caspase-3 proteins in HSC-3 cells increased significantly, and the expression of the p-EGFR, p-AKT, and Bcl-2 proteins decreased significantly, indicating that geniposide may inhibit oral squamous cell carcinoma through multi-target regulation of the PI3K-Akt signaling pathway.

However, it is worth noting that compared to conventional chemotherapeutic agents, the relatively high IC50 value of geniposide (2.766 mmol/L) may limit its clinical application. We are considering using nanotechnology [44,45,46,47], such as developing polymeric nanoparticles, micelles, or other nanomedicine formulations or reducing the drug particle size through nanotechnology, to enhance efficacy, mitigate toxicity, and improve utilization. Concerning molecular dynamics simulation, it is worth noting that longer simulation trajectories could enhance the analysis of system stability and strengthen credibility. In future studies, we will extend the simulation trajectories and conduct more comprehensive analyses. Furthermore, this study used a single cell line (HSC-3) for in vitro validation, which limits the findings. In subsequent work, we will extend to other oral squamous cell carcinoma cell lines, such as HSC-2, SCC-9, and CAL-27, and normal oral keratinocytes, alongside in vivo studies in animal models. This will further validate and elucidate the mechanism of geniposide in treating oral squamous cell carcinoma.

## 5. Conclusions

This experiment offers a novel concept and theoretical foundation for understanding how geniposide controls the occurrence and progression of OSCC. The efficacy component of *Gardenia jasminoides* J.Ellis, with homology of medicine and food from natural sources, provides an essential foundation for the future development and application of geniposide.

## Figures and Tables

**Table 1:** Top 10 targets of PPI network.

Rank	Name	Degree
1	EGFR	44
2	AKT1	40
3	SRC	38
4	HSP90AA1	36
5	PIK3R1	31
6	MMP9	28
7	ESR1	27
8	STAT1	23
8	HSP90AB1	23
9	CASP3	22
9	IGF1	22
10	ALB	20

## Data Availability

The data supporting this study can be obtained from the corresponding author.
